# Draft Genome Sequence of *Pseudomonas koreensis* CI12, a *Bacillus cereus* “Hitchhiker” from the Soybean Rhizosphere

**DOI:** 10.1128/genomeA.00570-17

**Published:** 2017-06-29

**Authors:** Gabriel L. Lozano, Juan I. Bravo, Jo Handelsman

**Affiliations:** Department of Molecular, Cellular and Developmental Biology, Yale University, New Haven, Connecticut, USA

## Abstract

*Pseudomonas koreensis* CI12 was coisolated with *Bacillus cereus* from a root of a soybean plant grown in a field in Arlington, WI. Here, we report the draft genome sequence of *P. koreensis* CI12 obtained by Illumina sequencing.

## GENOME ANNOUNCEMENT

*Pseudomonas koreensis* was first proposed as a novel *Pseudomonas* species to classify several isolates from Korean agricultural soils (1). Since then, strains worldwide have been identified with diverse capacities ranging from the production of antibacterial compounds (2) to the suppression of plant diseases caused by oomycete pathogens (3). Recently, whole-genome sequenced-based analyses designated *P. koreensis* as a defined phylogenomic group within the physiologically and genetically heterogeneous *Pseudomonas fluorescens* complex (4, 5). The *P. koreensis* group contains members that have been isolated from *Populus* root systems (6) and is, more generally, enriched with isolates recovered from diverse plants (7). Additionally, a comparative genomic analysis within the *P. fluorescens* complex showed an overrepresentation of traits related to plant-bacterium interactions in genomes from *P. koreensis* isolates (4).

*P. koreensis* CI12 was isolated as one of several microbial “hitchhikers” from *Bacillus cereus* cultures purified from field-grown soybean roots (8). These hitchhikers are bacteria that are not visible in colony-purified *B. cereus* cultures until 2 to 4 weeks of incubation at 4°C; although 3 to 5% of *B. cereus* isolates from soybean roots carry hitchhikers, the mechanism underlying the association is unknown. The classification of CI12 within the *P. fluorescens* complex was determined by independent phylogenetic reconstruction of the *gyrB*, *rpoD*, and *rpoB* genes, as has been described previously (4). *Pseudomonas koreensis* CI12 was selected as a model for studying bacterial interactions in the rhizosphere. *In vitro* growth of *P. koreensis* CI12 in root exudate is not significantly affected by the presence of *B. cereus*, but *P. koreensis* CI12 can impair the growth of other hitchhikers, which is in contrast to the hitchhikers’ growth enhancement by *B. cereus* (8).

The *P. koreensis* CI12 genome was sequenced on the Illumina MiSeq platform. A total of 8,588,279 paired-end reads of 300 bp from a library with an average insert size of 1 kb were generated. Low-quality sequences were trimmed using Trimmomatic (9), and the resulting sequences were then assembled using Velvet (10) and VelvetOptimiser. Contigs were ordered by Mauve (11) using the *P. fluorescens* Pf0-1 genome (12) as a reference, assembled manually by joining with a linker sequence of unknown nucleotide character “N,” and then gaps were filled with GapFiller (13). The resulting assembly was 6,622,028 bp, consisting of 16 contigs, with an *N*_50_ contig size of 608,098 bp.

We predict that sequencing new strains of *P. koreensis* will help delineate traits that may mediate its interactions with plant hosts and their associated microbiota. Furthermore, additional genomes belonging to members of the *P. fluorescens* complex may help define the phylogenomic groups and determine their relationship with ecophysiological groups of the complex.

### Accession number(s).

This whole-genome shotgun project has been deposited at DDBJ/ENA/GenBank under the accession no. MPLD00000000. The version described in this paper is the first version.
